# Ligand-Binding Affinity at the Insulin Receptor Isoform-A and Subsequent IR-A Tyrosine Phosphorylation Kinetics are Important Determinants of Mitogenic Biological Outcomes

**DOI:** 10.3389/fendo.2015.00107

**Published:** 2015-07-07

**Authors:** Harinda Rajapaksha, Briony E. Forbes

**Affiliations:** ^1^School of Biological Sciences, University of Adelaide, Adelaide, SA, Australia; ^2^Department of Medical Biochemistry, School of Medicine, Flinders University of South Australia, Bedford Park, SA, Australia

**Keywords:** insulin receptor, insulin analogs, IGF-II, mitogenic, metabolic, receptor internalization, intracellular signaling

## Abstract

The insulin receptor (IR) is a tyrosine kinase receptor that can mediate both metabolic and mitogenic biological actions. The IR isoform-A (IR-A) arises from alternative splicing of exon 11 and has different ligand binding and signaling properties compared to the IR isoform-B. The IR-A not only binds insulin but also insulin-like growth factor-II (IGF-II) with high affinity. IGF-II acting through the IR-A promotes cancer cell proliferation, survival, and migration by activating some unique signaling molecules compared to those activated by insulin. This observation led us to investigate whether the different IR-A signaling outcomes in response to IGF-II and insulin could be attributed to phosphorylation of a different subset of IR-A tyrosine residues or to the phosphorylation kinetics. We correlated IR-A phosphorylation to activation of molecules involved in mitogenic and metabolic signaling (MAPK and Akt) and receptor internalization rates (related to mitogenic signaling). We also extended this study to incorporate two ligands that are known to promote predominantly mitogenic [(His^4^, Tyr^15^, Thr^49^, Ile^51^) IGF-I, qIGF-I] or metabolic (S597 peptide) biological actions, to see if common mechanisms can be used to define mitogenic or metabolic signaling through the IR-A. The threefold lower mitogenic action of IGF-II compared to insulin was associated with a decreased potency in activation of Y960, Y1146, Y1150, Y1151, Y1316, and Y1322, in MAPK phosphorylation and in IR-A internalization. With the poorly mitogenic S597 peptide, it was a decreased rate of tyrosine phosphorylation rather than potency that was associated with a low mitogenic potential. We conclude that both decreased affinity of IR-A binding and kinetics of IR-A phosphorylation can independently lead to a lower mitogenic activity. None of the studied parameters could account for the lower metabolic activity of qIGF-I.

## Introduction

The insulin and insulin-like growth factor (IGF) system comprises the three highly similar ligands (insulin, IGF-I, and IGF-II). While insulin controls blood glucose levels, IGF-I promotes postnatal growth and IGF-II plays important roles during fetal development by promoting proliferation and differentiation in a range of different tissues. The importance of IGF-II is highlighted by the fact that its actions are tightly regulated by an IGF-II specific cation-independent mannose-6-phosphate/IGF2 receptor (IGF2R) that regulates circulating IGF-II levels by targeting it to lysosomal degradation (1). All three ligands act via a family of tyrosine kinase receptors including the insulin receptor (IR), type 1 IGF receptor (IGF-1R), and hybrid receptors (formed between the IR and IGF-1R). Insulin binds with high affinity to the IR to promote metabolic signaling and IGF-I and IGF-II act via the IGF-1R and hybrid receptors to promote mitogenic signaling, such as cell survival, growth, and proliferation (2, 3).

A series of *in vitro* and *in vivo* studies have shown that IGF-II also signals via one of the two IR isoforms arising from alternative splicing of the *IR* gene (exon 11− IR-A and exon 11+ IR-B)(4). Whereas the IR-B includes the 12 amino acids encoded by exon 11 (5) and its activation by insulin leads to metabolic actions, the IR-A binds both IGF-II and insulin with high affinity to promote mitogenic outcomes (6–8). Many cancer cells express both IGF-II and IR-A and the IGF-II/IR-A signaling pathway promotes cancer cell proliferation and survival (9–11). Cancer cells can use this pathway as an additional or alternate mitogenic pathway to signaling via the type-1 IGF receptor (IGF-1R), and can provide a mechanism by which cancer cells can become resistant to treatments targeting the IGF-1R (4, 12). An understanding of how mitogenic processes are activated downstream of the IR-A will ultimately lead to improved strategies to inhibit this signaling pathway and will potentially provide novel cancer treatments.

In exploring the role of the IGF-II/IR-A signaling pathway in promoting cancer cell proliferation, survival, and migration, Belfiore and colleagues (13–15) demonstrated that IGF-II promotes a signaling pattern that differs from insulin while also sharing some common signaling pathways. This suggests that the subtle differences in the way in which ligands interact with the IR-A can influence the resultant downstream signaling events, although the details of the mechanisms are still to be fully understood. In support of this hypothesis, our laboratory and others have observed that in some cases different IGF and insulin analogs bind the IR with similar affinities and yet promote different biological outcomes (7, 16, 17).

Binding of ligand to the IR extracellular domain induces a conformational change that is transduced to the intracellular tyrosine kinase domain resulting in activation and tyrosine autophosphorylation of the receptor. Nine of the 13 IR intracellular domain tyrosine residues, including Y960 of the juxtamembrane domain (JM), Y1146, Y1150, and Y1151 of the kinase activation loop, and Y1316 and Y1322 of the carboxy-terminal tail (IR-A numbering), are phosphorylated under various conditions (18). Subsequently, signaling molecules including insulin receptor substrates (IRS), SHC, APS, and Grb proteins are recruited and activation of the downstream pathways follows. The two main pathways activated downstream of the IR are the PI3 kinase (PI3K) and protein kinase B PKB/Akt pathways. The PkB/Akt pathway is involved in promoting metabolic processes, such as glucose uptake into muscle and adipose, as well as mitogenic processes involving protein translation and cell cycle progression, whereas the MAPK pathway is mostly involved in mitogenic signaling (4, 19).

There are several factors that could result in different signaling outcomes promoted by two ligands interacting with the IR-A with similar affinities. These include differences in ligand residence time on the receptor and differences in receptor internalization rates promoted by each ligand (20). Slow dissociation of ligands from the receptor can cause sustained activation of the IR and promotes phosphorylation of SHC (21) with a concomitant increased mitogenic response compared to insulin (22, 23). This response is linked to IR internalization, whereby ligands that promote phosphorylation of SHC and MAPK (24) promote IR internalization [including the IR-A (25)]. Molecules involved in mitogenic signaling such as Grb2, SHC, and MAPK have been found to co-localize with the endosomal IR (20) and inhibition of IR internalization significantly reduces insulin-induced Shc and MAPK phosphorylation (26). This suggests that internalization is important for the phosphorylation of Shc and MAPK. In contrast, the rapid response following IR activation of IRS-1 and Akt phosphorylation that leads to metabolic signaling outcomes is not dependent on internalization (26, 27).

The first step leading to two ligands promoting different biological outcomes despite having similar receptor binding affinities may relate to ligand-induced receptor phosphorylation. Indeed, mutation of a single IGF-II residue (Glu12) to Lys disproportionately affected the level of IR-A phosphorylation and subsequent ability to activate Akt (28). In order to explore this further and understand how IGF-II promotes IR-A-mediated activation of different signaling pathways when compared to insulin, we decided to measure the phosphorylation of IR-A tyrosine residues in response to these ligands. This pattern of IR-A phosphorylation was then correlated to the ability to stimulate receptor internalization, to activate the Akt and MAPK pathways and to promote DNA synthesis (mitogenic activity).

In addition, two insulin mimetic peptides were studied in parallel with IGF-II and insulin, as they are examples of ligands with the same affinity as insulin for the IR-A, but promote either predominantly mitogenic or metabolic signaling outcomes. The first is an insulin agonist peptide selected by phage display (S597) that binds the IR-A with equal affinity to insulin and yet does not activate mitogenic signaling while retaining the ability to promote metabolic signaling (16, 29). S597 is a 3.7 kDa peptide (Ac-SLEEEWAQIECEVYGRGCPSESFYDWFERQL-amide), which is believed to adopt a two-helix structure partially representing the structure of insulin, thus allowing it to bind to the receptor (16, 30–32). The second analog (His^4^, Tyr^15^, Thr^49^, Ile^51^) IGF-I (quadruple IGF-I or qIGF-I), binds the IR-A with only twofold lower affinity than insulin and yet is at least 10-fold less potent in its ability to stimulate metabolic activity as measured by glycogen synthesis (33). qIGF-I is, however, equipotent with insulin in stimulating mitogenic activity (7).

By studying the abilities of insulin, IGF-II, S597, and qIGF-I to activate IR phosphorylation at specific sites and correlating this to their abilities to promote receptor internalization and subsequent metabolic and mitogenic actions, we have been able to provide some insight into how different ligands elicit different biological activities through the IR. The results highlight the need to study not only binding affinities but also the kinetics of receptor activation when trying to explain the mechanism by which different ligands can stimulate different biological actions.

## Materials and Methods

Insulin was purchased from Lyppard Australia Pty Ltd. IGF-II and (His^4^, Tyr^15^, Thr^49^, Ile^51^) IGF-I (qIGF-I) were produced in-house as described by Ref. (7) and the S597 peptide was provided by Dr. L. Schäffer, Novo Nordisk Denmark. Hybridoma cells expressing antibodies specific for the IR alpha subunit (83-7) and beta subunit (CT-1) were a kind gift from Siddle (34, 35). Anti-phospho IGF-1R/IR Y1158, Y1161, Y1162 (p3Y = pY1146, pY1150, pY1151 IR-A numbering), anti-phospho IR Y960 (pY960), and anti-beta tubulin were from Invitrogen (Life Technologies, Mulgrave, VIC, Australia). Anti-phospho IR Y1316 and anti-phospho Y1322 and the Pathscan^®^ Multiplex Western Cocktails were purchased from Cell Signaling Technology Inc. (Danvers, MA, USA). Cy3 AffiniPure Donkey Anti-Mouse IgG and Cy5 AffiniPure Donkey Anti-Rabbit IgG were purchased from Jackson/Abacus ALS. Europium-labeled anti-phosphotyrosine antibody (Eu-pY20), europium-labeled-streptavidin (Eu-SA) and [^3^H] thymidine were purchased from PerkinElmer Life Sciences. hIR-A overexpressing R^−^ fibroblast cells (derived from IGF-1R knockout mouse embryonic fibroblasts) were produced by (6). hIR-A overexpressing L6 myoblasts were kindly provided by Dr. B. F. Hansen (Novo Nordisk A/S, Denmark). Protease inhibitor cocktail and NHS-Biotin were from Sigma.

### Competition binding assays

IR-A binding was measured essentially as described by Ref. (6). Briefly, R^–^IR-A cells were serum-starved for 4 h before lysis in lysis buffer [20 mM HEPES, 150 mM NaCl, 1.5 mM MgCl_2_, 10% (v/v) glycerol, 1% (v/v) Triton X-100, 1 mM EGTA, and 1 mM phenylmethylsulfonyl fluoride, pH 7.5] for 1 h at 4°C. Lysates were centrifuged for 10 min at 2,200 × *g*, then 100 μl lysate was added per well to a white Greiner Lumitrac 600 96-well plate previously coated with anti-IR antibody 83-7 (250 ng/well in bicarbonate buffer pH 9.2) (34). Approximately 500,000 fluorescent counts of Eu-insulin (prepared in-house) was added to each well along with increasing concentrations of unlabeled competitor in a final volume of 100 μl and incubated for 16 h at 4°C. Wells were washed four times with 20 mM Tris, 150 mM NaCl, and 0.1% (v/v) Tween 20 (TBST). Then 100 μl per well DELFIA enhancement solution (PerkinElmer Life Sciences) was added. Time-resolved fluorescence was measured using 340 nm excitation and 612 nm emission filters with a BMG Lab Technologies Polarstar fluorometer (Mornington, VIC, Australia). Assays were performed in triplicate at least three times.

### Kinase receptor activation assay

Insulin receptor-A phosphorylation was detected essentially as described by Ref. (6). Briefly, R^–^IR-A cells (5 × 10^4^ cells/well) were plated in a 96-well flat-bottom plate and grown overnight at 37°C, 5% CO_2_. Cells were starved in serum-free medium (SFM) for 4 h before treatment with insulin, IGF-II, qIGF-I, or S597 in 100 μl of Dulbecco’s minimal essential medium with 1% bovine serum albumin for 10 min or in a time course (0, 2, 5, 8, 12, 20, 30 min) at 37°C, 5% CO_2_. Cells were lysed with ice-cold lysis buffer containing 2 mM Na_3_VO_4_ and 100 mM NaF, and receptors were captured onto white Greiner Lumitrac 600 96-well plates pre-coated with anti-IR antibody 83-7 (500 ng/well) (34) and blocked with 20 mM Tris, 150 mM NaCl, and 0.1% (v/v) Tween 20 (TBST)/0.5% bovine serum albumin. Following overnight incubation at 4°C, the plates were washed three times with TBST. Phosphorylated receptor was detected by incubation with EU-pY20 (76 ng/well) at room temperature for 2 h. Wells were washed four times with TBST, and time-resolved fluorescence was detected as described above. Assays were performed in triplicate at least three times.

### Western immunoblotting

R^−^ IR-A cells were treated with 10 or 100 nM ligand for 10 min or 10 nM ligand in a time course (0, 2, 5, 8, 12, 20, 30 min) after a 4 h serum starvation with DMEM (1% BSA). Cells were lysed in the lysis buffer described above with freshly added 0.1% (v/v) protease inhibitor cocktail, 2 mM Na_3_VO_4_ and 100 mM NaF. Protein concentration was determined by bicinchoninic acid (BCA) assay (Pierce, Life Technologies). Lysates (35 μg) were subjected to reducing SDS-PAGE (7.5 or 12% glycine gel) and transferred to Hybond LFP transfer membrane (GE Healthcare). Blots were probed with anti-phospho IR Y1146, Y1150, Y1151 (p3Y), anti-phospho IR Y960, anti-phospho IR Y1316, anti-phospho IR Y1322, anti-IR beta-subunit antibody CT-1, pathscan^®^ Multiplex Western Cocktail or anti-tubulin following the manufactures’ recommendations and anti-mouse cy3 and anti-rabbit cy5 were used as secondary antibodies. Finally, the blots were scanned using the Typhoon TRIO Variable mode Imager Amersham Biosciences. Blots were quantitated using ImageJ software V1.44. Blots were performed at least three times.

### Receptor internalization assay

The internalization of IR-A was assessed using an ELISA assay as described in Ref. (36) with modifications. R^−^ IR-A cells (2 × 10^5^ cells/well) were plated in a six-well plate and grown overnight at 37°C, 5% CO_2_. Cells were starved in SFM for 4 h before treatment with increasing concentrations of insulin, IGF-II, qIGF-I, or S597 in 500 μl of Dulbecco’s minimal essential medium with 1% BSA for 30 min or in a time course (0, 2, 5, 8, 12, 20, 30 min) at 37°C, 5% CO_2_. After the stimulation, the medium was aspirated, and cell surface proteins were biotinylated with NHS-Biotin (0.5 mg/ml) in ice-cold PBS (2.68 mM KCl, 1.46 mM KH_2_PO_4_, 136.9 mM NaCl, 8.1 mM Na_2_HPO_4_, pH7.4). After 15 min, plates were washed with three gentle ice-cold TBS (20 mM Tris, 150 mM NaCl) washes and lysed in the lysis buffer described above. The receptors were captured onto white Greiner Lumitrac 600 96-well plates pre-coated with anti-IR antibody CT-1 (250 ng/well) and blocked with 0.5% BSA in TBST. Following 1 h incubation at room temperature, the plates were washed three times with TBST and biotinylated IR was detected following incubation with 76 ng/ml Eu-SA at room temperature for 1 h. Plates were washed three times with TBST and time-resolved fluorescence was detected as described above. Assays were performed in triplicate at least three times.

### DNA synthesis assay

DNA synthesis was carried out as described in Ref. (7). The rate of proliferation of R^−^ IR-A cells was such that a sufficient difference between stimulated and unstimulated cells was not easily achieved. Therefore, the well-characterized L6 rat skeletal myoblasts stably overexpressing human IR-A were used. Briefly, L6 rat skeletal myoblasts (1.5 × 10^4^ cells/well), stably overexpressing human IR-A, were plated in a 96-well flat-bottom plate and grown overnight at 37°C, 5% CO_2_. Cells were starved in SFM for 4 h before treatment with insulin, IGF-II, qIGF-I, or S597 with increasing ligand concentrations for 19 h in Dulbecco’s minimal essential medium with 1% bovine serum albumin. The cells were pulsed with 0.14 μCi/well [^3^H] thymidine for 4 h and harvested onto glass fiber filters (Millipore^®^) using a MICRO 96™ Skatron harvester (Molecular Devices). The filters were counted in a Wallac MicroBeta counter (PerkinElmer Life Sciences).

### Statistical analyses

Two-way ANOVA followed by Tukey’s *post hoc* was used for statistical analysis of blots. Significance was accepted at *p* < 0.05.

## Results

### IR-A binding affinities

Binding affinities of insulin, IGF-II, qIGF-I, and S597 for IR-A were compared in competitive binding assays. Insulin bound to the IR-A with an EC_50_ of 1.57 nM, whereas IGF-II bound with an IC_50_ of 15.21 nM (Table 1), a 10-fold lower affinity than insulin. qIGF-I bound IR-A with a threefold lower affinity than insulin, and of all the ligands, S597 had the highest affinity for the IR-A with an IC_50_ of 0.75 nM (twofold higher affinity than insulin). These results correlate with previous reports (7, 29).

**Table 1:** **Inhibition of europium-labeled insulin (Eu-insulin) for binding to the IR-A**.

	Insulin	IGF-II	qIGF-I	S597
IC_50_ (nM)	1.57 ± 0.33	15.21 ± 0.18	4.88 ± 0.8	0.75 ± 0.16
Rel. IC_50_ (%)	100	10.3	32.2	209

*The concentration at which 50% of Eu-insulin binding was inhibited by each peptide (IC_50_ nM) is given. The IC_50_ relative to that of insulin is also shown. Values are the means ± SEM of three independent experiments*.

### IR-A tyrosine phosphorylation

#### IR-A Total Tyrosine Phosphorylation Induced by Different Ligand Concentrations

**Figure 1:**
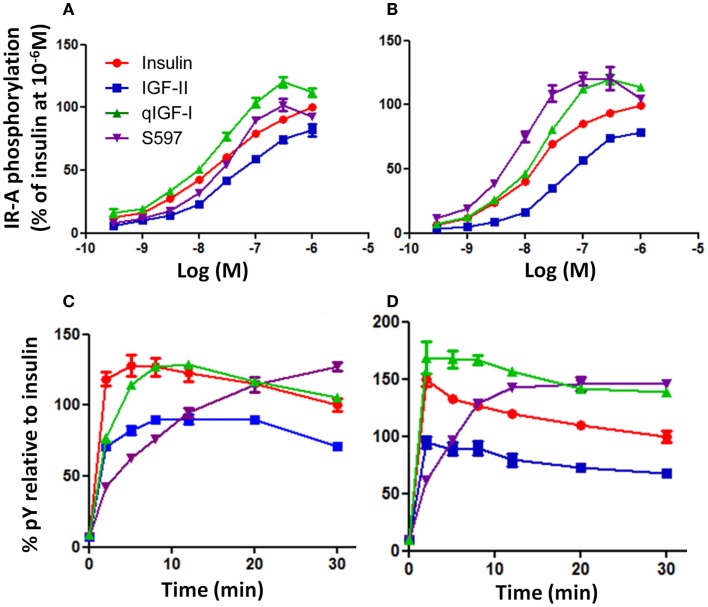
**Phosphorylation of IR-A in response to insulin, IGF-II, qIGF-I, and S597**. R^−^ IR-A cells were incubated with increasing concentrations of insulin (●), IGF-II (■), qIGF-I (▲), or S597 (▼) for 10 min **(A)** or 30 min **(B)** following 4 h serum starvation. Solubilized IR-A was immunocaptured, and phosphorylated tyrosines were detected with Eu-PY20. The receptor phosphorylation is expressed as a percentage of the phosphorylation induced by insulin at 10^−6^ M. Basal phosphorylation in serum-starved cells was 2% (not shown). In addition, R^−^ IR-A cells were incubated with 10 nM **(C)** or 100 nM **(D)** ligand for increasing times up to 30 min. The receptor phosphorylation is expressed as a percentage of the phosphorylation induced by insulin at *t* = 30 min. The data points are means ± SEM of three assays with each concentration measured in triplicate. Error bars are shown when greater than the size of the symbols.

In order to measure the ability of the ligands to activate IR-A phosphorylation upon binding R^−^ IR-A cells were stimulated with a series of concentrations (0.3–1000 nM) of insulin, IGF-II, qIGF-I, or S597 and the potency of each ligand to phosphorylate IR-A was measured using a kinase receptor activation (KIRA) assay. The highest level of total tyrosine phosphorylation induced by insulin was achieved at 1000 nM after 10 min stimulation, although phosphorylation had not reached a maximum at this concentration (Figure 1A). IGF-II, which had a 10-fold lower affinity for IR-A, was less potent than insulin in stimulating IR-A phosphorylation. There was a rightward shift in EC_50_ and the highest level of total tyrosine phosphorylation induced by IGF-II was achieved at 1000 nM but maximal phosphorylation was not reached (Figure 1A; Table 2). Unexpectedly, at 10 min stimulation, qIGF-I, which had a threefold lower affinity for IR-A than insulin, was the most potent ligand in stimulating total phosphorylation, with maximal tyrosine phosphorylation achieved at 300 nM and higher concentrations resulted in less than maximal phosphorylation. S597, which had a twofold higher affinity for IR-A, induced a similar total tyrosine phosphorylation to insulin after 10 min stimulation but maximal IR-A tyrosine phosphorylation was achieved at 300 nM. Generally, the pattern of phosphorylation induced by insulin, IGF-II, and qIGF-I after 30 min stimulation (Figure 1B; Table 2) was similar to that seen after 10 min stimulation (Figure 1A). Interestingly, however, S597 was the most potent ligand at 30 min stimulation with a lower EC_50_ and a greater maximal response than qIGF-I, insulin, or IGF-II.

**Table 2:** **Summary of IR-A phosphorylation, internalization, and downstream signaling promoted by insulin, IGF-II, qIGF-I, and S597**.

Potencies relative to insulin													
	IR-A affinity^a^	Total IR pY (10 min)^b^	Total IR pY (30 min)^c^	IR residue-specific phosphorylation (10 min)^d^				IR-A internalization (30 min)^e^	pAKT (S473) (10 min)^f^	pMAPK (10 min)^g^	Rate of phosph.	Mitogenic activity^h^	Metabolic activity
				p3Y	pY960	pY1316	pY1322						
Insulin	1.0	1.0	1.0	1.0	1.0	1.0	1.0	1.0	1.0	1.0	Rapid	1.0	1.0
qIGF-I	0.3	1.5	1.0	1.1	1.5	1.3	1.3	1.1	1.1	1.0	Rapid	0.8	0.02 (7)
IGF-II	0.1	0.5	0.3	0.5	0.5	0.5	0.3	0.6	0.8	0.6	Rapid	0.2	0.4 (37)
S597	2.0	0.9	2.7	0.5	0.4	0.6	0.4	0.2	0.7	0.3	Gradual	0.06	1.0 (16)

*Potencies of qIGF-I, IGF-II, and S597 are presented as relative values compared to insulin. Insulin binds IR-A with a high affinity and promotes rapid total receptor phosphorylation, residue-specific phosphorylation (rate of phosph.), and internalization. qIGF-I binds IR-A with threefold less affinity but at 10 min the total receptor phosphorylation and IR residue-specific phosphorylation were slightly higher than in response to insulin. Akt and MAPK phosphorylation, internalization, and mitogenic activities induced by qIGF-I were similar to insulin. However, qIGF-I was significantly lower in metabolic potency (33). IGF-II bound to IR-A with a 10-fold lower affinity compared to insulin and resulted in a similar decrease in mitogenic potency. Despite this, surprisingly, IGF-II induced slightly higher responses of 3Y, Y960, Y1316, AKT (S473), MAPK phosphorylation, IR-A internalization (30–80% of insulin) and metabolic (37) potency than expected. In contrast, S597 binds to IR-A with twofold higher affinity compared to insulin. S597 induced a gradual increase in total receptor phosphorylation, which translated to a similar IR-A phosphorylation to insulin after 10 min but a significantly higher potency at 30 min. At the residue-specific level, 3Y, Y1316, and Y1322 phosphorylation induced by S597 was 40–60% of the response to10 nM insulin. AKT (S473) phosphorylation induced by S597 was similar to insulin, while MAPK phosphorylation was approximately 30% of that induced by 10 nM insulin. Strikingly, the phosphorylation rate of IR-A and downstream signaling molecules was much slower in response to S597 than to all other ligands. The mitogenic potency of S597 was significantly lower (16-fold) than that of insulin, while remaining equipotent in metabolic activity (16). Notably IR-A internalization rates stimulated by all ligands correlated well with MAPK phosphorylation. Where these were decreased (in response to IGF-II and S597), mitogenic potency was also decreased*.

*^a^Affinity: EC50 insulin = 1.6 nM, from Table 1*.

*^b^IR total phosphorylation after 10 min stimulation: EC50 insulin = 24 nM, from Figure 1A*.

*^c^IR total phosphorylation after 30 min stimulation: EC50 insulin = 10 nM, from Figure 1B*.

*^d^IR residue-specific phosphorylation, from Figure 3*.

*^e^IR internalization at 30 min by 10 nM ligand: surface/total IR insulin = 80%, from Figure 5A*.

^f^pAKT (S473) and

**Figure 2:**
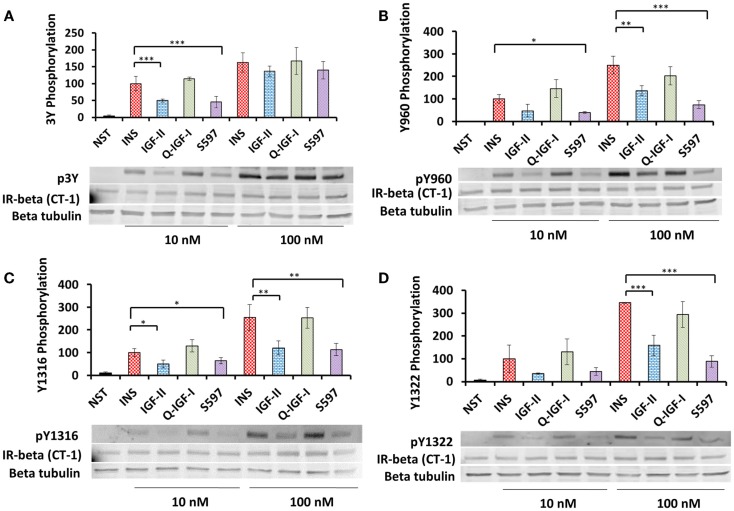
**Induction of p3Y (A), pY960 (B), pY1316 (C), and pY1322 (D) phosphorylation by insulin, IGF-II, qIGF-I, and S597**. Serum-starved R^−^ IR-A cells were treated with 10 or 100 nM insulin (INS), IGF-II, qIGF-I, or S597 for 10 min. Whole-cell lysates were prepared and subjected to SDS-PAGE and then immunoblotted for phosphorylated 3Y, Y960, Y1316, and Y1322. Representative blots are shown in the lower panels. In upper panels, quantitations of three averaged independent experiments ± SEM are shown as a column graph. Phosphorylation levels are expressed as a percentage of the level detected when cells were stimulated with 10 nM insulin (100%). β-tubulin and IR beta were probed as loading controls. NST = non-stimulated. ***p value <0.001, **p value 0.001–0.01, *p value 0.01–0.05 when compared between 10 and 100 nM for the same stimulating ligand.

*^g^pMAPK stimulated by 10 nM ligand: insulin was denoted 100%, from Figures 2 and 4*.

*^h^Mitogenic activity: EC50 insulin = 0.95 nM, from Figure 5B*.

#### Time-Dependent Total Tyrosine Phosphorylation

The observation that the relative potencies of S597 changed with time prompted us to undertake a time course analysis of phosphorylation. R^−^ IR-A cells were treated with 10 or 100 nM ligand over a time course of 30 min (Figures 1C,D). At both concentrations, insulin induced rapid total tyrosine phosphorylation of IR-A, and a maximum was reached after 2–5 min (Figures 1C,D). IGF-II also induced a rapid total tyrosine phosphorylation of IR-A, and reached a maximum after 8–12 min (Figure 1C). The level of total tyrosine phosphorylation induced by IGF-II gradually decreased to 71% of that stimulated by insulin at 30 min. The total tyrosine phosphorylation profile observed after stimulating with 10 nM qIGF-I was similar to 10 nM insulin although a maximum was reached after 12 min and thereafter gradually decreased to the same level as insulin at 30 min. In contrast, stimulation with 10 nM S597 did not initiate a rapid total tyrosine phosphorylation of IR-A. Instead, a gradual increase of total tyrosine phosphorylation was observed, with the highest level of phosphorylation measured at 30 min (Figure 1C).

Generally, the time-dependent phosphorylation profile induced by 100 nM insulin, IGF-II, qIGF-I, and S597 (Figure 1D) was similar to that seen at 10 nM (Figure 1C). However, 100 nM qIGF-I appears more potent than 100 nM insulin with time, with a significantly greater response (140%) at 30 min. Again 100 nM S597 induced a gradual increase in phosphorylation but at this concentration, reached a maximum after 20 min stimulation, which was also greater than (140%) the response to 100 nM insulin at 30 min.

In summary, each of the four ligands exhibited different potencies and kinetics of IR-A activation (see Table 2). IGF-II was the least potent, most likely due to its relatively low affinity for the IR-A. However, the kinetics of IGF-II and qIGF-I activation were similar to insulin with a rapid response, which then gradually decreased with time. qIGF-I induced a significantly higher level of IR-A total tyrosine phosphorylation compared to insulin. Out of all the ligands tested, S597 was unique in its ability to induce IR-A total tyrosine phosphorylation, as it promoted a gradual increase of receptor total tyrosine phosphorylation, suggesting a different mode of IR-A activation by S597 compared to insulin, IGF-II, or qIGF-I.

#### Residue-Specific Phosphorylation

In order to investigate whether differences in total tyrosine phosphorylation were caused by differences in residue-specific tyrosine phosphorylation induced by each ligand phosphorylation of residues Y960 in the JM, Y1146, Y1150, and Y1151 in the kinase domain, IR Y1316 and Y1322 in the C-terminal tail was measured by immunoblotting (IR-A numbers). Insulin at 10 nM was twofold more potent than IGF-II and S597 in inducing 3Y phosphorylation (Figure 2A; Table 2). In contrast, qIGF-I was as potent as insulin in stimulating 3Y phosphorylation (Figure 2A). Insulin at 100 nM induced a twofold greater level of phosphorylation compared to 10 nM insulin. However, no significant difference was seen between the four ligands at 100 nM in their ability to stimulate 3Y phosphorylation (Figure 2A), suggesting the upper limit of detection had been reached.

A similar pattern was seen for phosphorylation of Y960 (Figure 2B; Table 2), Y1316 (Figure 2C; Table 2), and Y1322 (Figure 2D; Table 2), where both 10 nM insulin and qIGF-I were equipotent in stimulating phosphorylation and on the other hand IGF-II and S597 were twofold less potent. At 100 nM, insulin stimulated 2.5-fold greater phosphorylation of these residues compared to 10 nM insulin. However, unlike the 3Y response (Figure 2A), a difference in relative potencies of each ligand to stimulate Y960 phosphorylation was evident at 100 nM ligand concentration (Figure 2B). Insulin and qIGF-I (100 nM) were similar in their potency to induce Y960 (Figure 2B), Y1316 (Figure 2C), and Y1322 (Figure 2D) phosphorylation, whereas IGF-II was ~2-fold less potent and S597 induced threefold less phosphorylation compared to insulin. This is in stark contrast to the ability of S597 to stimulate total phosphorylation (Figures 1A,D) where 100 nM S597 was equipotent to insulin at 10 min stimulation. To investigate whether this is due to a time effect, the time-dependent residue-specific phosphorylation was studied.

#### Time-Dependent Residue-Specific Phosphorylation

**Figure 3:**
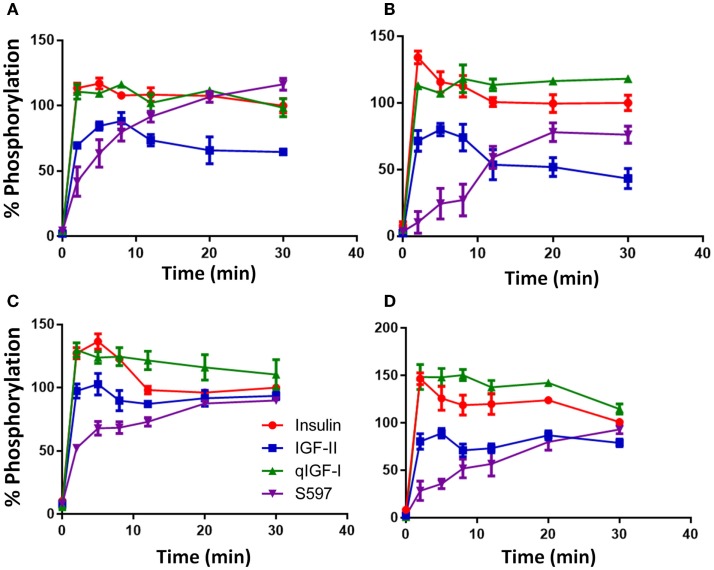
**Time course of phosphorylation of p3Y (A), pY960 (B), pY1316 (C), and pY1322 (D) after stimulating with insulin, IGF-II, qIGF-I, or S597**. Serum-starved R^−^ IR-A cells were treated with 10 nM insulin (●), IGF-II (■), qIGF-I (▲), or S597 (▼) in a time course of 30 min. Whole-cell lysates were prepared and subjected to SDS-PAGE and then immunoblotted for phosphorylated 3Y, Y960, Y1316, and Y1322. Response to ligand stimulation is expressed as the percentage phosphorylation stimulated by 10 nM insulin for 30 min (100%). Basal phosphorylation in the presence of serum-free medium is shown at *t* = 0 min. Data are normalized to the IR-beta loading control. Quantitation of three independent experiments ± SEM is shown. Error bars are shown when greater than the size of the symbols.

Insulin (10 nM) induced a rapid phosphorylation of 3Y and maximum phosphorylation was reached after 2 min stimulation with the level of p3Y gradually decreasing thereafter over the 30 min time course (Figure 3A). Response to the other ligands was expressed relative to the level of insulin phosphorylation remaining at 30 min (designated 100%). Similar to insulin, qIGF-I also induced maximum 3Y phosphorylation after 2 min and thereafter the level of 3Y phosphorylation gradually reduced and was the same as the response to 10 nM insulin at 30 min. IGF-II (10 nM) induced maximal 3Y phosphorylation after 8 min and this maximum (90%) was lower than for insulin. With time, 3Y phosphorylation stimulated by IGF-II declined to 65% of the response to insulin at 30 min. In contrast, S597 induced a gradual increase of 3Y phosphorylation, reaching 116% after 30 min without reaching maximal response (Figure 3A). This gradual increase in 3Y phosphorylation mirrored what was seen in the total phosphorylation KIRA time course (Figure 1C).

The relative kinetics and potencies of the four ligands in stimulating phosphorylation of Y960 (Figure 3B) and the two C-terminal tyrosines Y1316 (Figure 3C) and Y1322 (Figure 3D) was similar to the 3Y phosphorylation profile (Figure 3A). Insulin and qIGF-I induced a rapid increase in phosphorylation of these residues and remained steady up to 30 min. Similar to p3Y, IGF-II was less potent than insulin. The most interesting differences in residue-specific phosphorylation were seen with S597, which stimulated a slower and more gradual increase in Y960 phosphorylation with time, reaching a maximum after 20 min stimulation, which was 80% of the response with 10 nM insulin after 30 min (Figure 3B). Unlike in experiments measuring 3Y phosphorylation, S597 was not as potent as insulin or qIGF-I in stimulating Y960 phosphorylation but was more potent than IGF-II. In contrast, in the case of pY1316 and pY1322 (Figures 3C,D), all ligands reached a similar level of phosphorylation at 30 min, although S597 again promoted a more gradual increase in phosphorylation over time.

### Activation of AKT and MAPK

**Figure 4:**
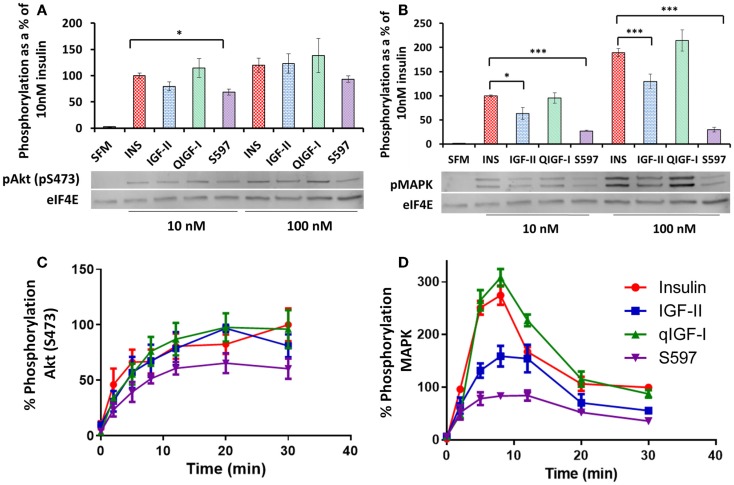
**Phosphorylation of AKT (pS473) and MAPK**. Serum-starved R^−^ IR-A cells were treated with 10 or 100 nM insulin (INS), IGF-II, qIGF-I, or S597 for 10 min. Whole-cell lysates were immunoblotted for phosphorylated pAKT (pS473) **(A)** and pMAPK **(B)**. Representative blots are shown in the lower panels. Relative phosphorylation levels are expressed as a fraction of the level detected when cells were stimulated with 10 nM insulin for 10 min. Basal phosphorylation in the presence of serum-free medium (SFM) is shown. Data are normalized to the loading control eIF4E. ***p value < 0.001, **p value 0.001–0.01, *p value 0.01–0.05. R^−^ IR-A cells were also treated with 10 nM insulin (●), IGF-II (■), qIGF-I (▲), or S597 (▼) over 30 min. Whole-cell lysates were immunoblotted for phosphorylated pAKT (pS473) **(C)** and pMAPK **(D)**. Relative phosphorylation levels are expressed as a percentage of the level detected when cells were stimulated with 10 nM insulin for 30 min (100%). In each case, the loading control eIF4E was used. Graphs represent the average of three independent experiments ± SEM. Error bars are shown when greater than the size of the symbols.

The abilities of the four ligands to activate AKT (S473) and MAPK were measured in R^−^ IR-A cells stimulated with 10 or 100 nM ligand for 10 min by immunoblotting. The level of AKT (S473) or MAPK phosphorylation induced by 10 or 100 nM insulin was designated as 100% above basal, respectively (Figures 4A,B; Table 2). Insulin and qIGF-I were equipotent in stimulating AKT (S473) and MAPK phosphorylation at 10 and 100 nM. S597 (*p* = 0.01–0.05 at 10 nM) and IGF-II (not significant) appeared to be slightly less potent than insulin and qIGF-I in stimulating pAkt activation (Figure 4A), whereas they were significantly less potent in promoting pMAPK activation at both concentrations (Figure 4B). In fact, 100 nM S597 was >80% less potent than insulin in activating MAPK.

As significantly different rates of IR-A phosphorylation were observed with S597 compared to insulin, we hypothesized that the lower level of MAPK phosphorylation induced by S597 may be due to different kinetics of S597-activated MAPK phosphorylation compared to insulin. Therefore, a time course of AKT and MAPK phosphorylation was studied after stimulating R^−^ IR-A cells with 10 nM insulin, IGF-II, qIGF-I, or S597. The rate of AKT (S473) phosphorylation induced by S597 was not statistically significantly different to the other ligands although the trend was for a slower rate of activation (Figure 4C). Interestingly, although stimulating with all four ligands lead to a sustained level of AKT (S473) phosphorylation, MAPK phosphorylation was transient (Figure 4D). The maximum MAPK phosphorylation in response to all four ligands was achieved after 8 min stimulation and in each case it decreased to approximately 50% of the maximum response after 30 min stimulation. Insulin and qIGF-I induced the same response whereas IGF-II and S597 were significantly less potent [~50 and 28%, respectively (*p* < 0.01)].

### IR-A internalization

**Figure 5:**
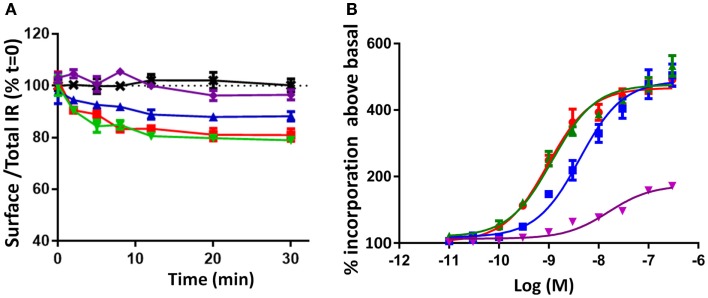
**Time course of IR-A internalization (A) and mitogenic potency measured by [^3^H] thymidine incorporation (B) induced by insulin, IGF-II, qIGF-I, and S597**. **(A)** Serum-starved R^−^ IR-A cells were treated with 10 nM ligand in a time course of 30 min. The cell surface proteins were biotinylated and IR-A was immunocaptured after cell lysis. The immunocaptured IR-A was probed with Eu-streptavidin and biotinylated IR-A was measured by time-resolved fluorescence. Internalization was measured by comparing the proportion of IR-A on the cell surface (biotinylated) relative to the proportion in the presence of serum-free medium (SFM = 100%, black dotted line). The data points are the mean ± SEM of at least three separate experiments with each point performed in triplicate. Error bars are shown when greater than the size of the symbols. **(B)** L6 rat muscle myoblasts overexpressing the IR-A were treated with increasing concentrations of insulin (●), IGF-II (■), qIGF-I (▲), or S597 (▼). The results are illustrated as percentage incorporation of [^3^H] thymidine above basal (no stimulation). Curves are plotted using the average of three assays ± SEM with each concentration measured in triplicate, and are analyzed using a sigmoidal dose–response curve fit with variable slope (Graphpad Prism V5.04). Error bars are shown when greater than the size of the symbols. The EC_50_ was determined for insulin (0.8 ± 0.17 nM), IGF-II (4.4 ± 0.4 nM), and qIGF-I (1.15 ± 0.01 nM) but not for S597.

Receptor internalization has previously been shown to influence IR mitogenic signaling (20). To understand the kinetics of IR-A internalization, time-dependent IR-A internalization was studied after stimulating R^−^ IR-A cells with 10 nM of each ligand over 30 min (Figure 5A). Insulin and qIGF-I (both 10 nM) were equipotent in inducing rapid IR-A internalization until 10 min (Figure 5A; Table 2), Thereafter, the level of surface receptor remained constant at 80% of total IR-A. IGF-II also rapidly induced IR-A internalization, but was less potent compared to insulin, as was observed by Morcavallo et al. (38). After 10 min, a constant level of IR-A was observed on the cell surface after stimulating with IGF-II (90% of total IR-A). S597, on the other hand, did not induce internalization of IR-A (Figure 5A), again supporting the observation by Jensen et al. (16). This lack of ability of S597 to induce IR-A internalization was in contrast to its high affinity for the IR-A.

### Biological activities promoted by insulin, IGF-II, qIGF-I, and S597 binding to the IR-A

Prior to this study, there was some discrepancy in the literature regarding the relative potencies of insulin and IGF-II in stimulating mitogenic actions (7, 37). Here, we confirmed that insulin and qIGF-I were equipotent in inducing DNA synthesis (EC_50_ = 0.83 and 1.15 nM respectively), whereas IGF-II was 5.5-fold less potent than qIGF-I (EC_50_ 4.4 nM) (Figure 5B), as reported by Gauguin et al. (7). In contrast, S597 had a significantly lower potency in inducing DNA synthesis [confirming the report by Jensen et al. (16)]. Previously, it was shown that IGF-II is less potent than insulin in stimulating metabolic activity (37), whereas qIGF-1 is 10-fold less potent (7) and S597 is equipotent to insulin (16).

## Discussion

With the aim to understand mechanisms underlying different biological activities promoted by different ligands via the same receptor, we compared the four ligands, insulin, IGF-II, qIGF-I, and the S597 peptide, for their ability to bind the IR-A and activate downstream signaling pathways. The receptor binding results demonstrated that the relative affinities of all four ligands are S597 > insulin > qIGF-I > IGF-II, with a 20-fold difference between S597 and IGF-II. These relative binding affinities correlate well with those in previous reports (6, 7, 29), although absolute binding affinities of S597 have not to our knowledge formally been reported in the literature.

The question then arose whether the resultant IR-A activation on R^−^ IR-A cells relates directly to these binding affinities. When total tyrosine phosphorylation was measured (pY20 in KIRA assay, Figure 1), the relative potencies correlated reasonably well with the IR-A binding affinities (Table 1). IGF-II was less potent than insulin in stimulating total IR-A tyrosine phosphorylation, as was similarly reported by Frasca et al. (37), and qIGF-I was slightly more potent than insulin (Figures 1A,B). Interestingly, in our study, the S597 peptide was equipotent to insulin in stimulating IR-A tyrosine phosphorylation. This is in contrast to the report by Jensen et al. (16), which showed by Western blotting (using 4G10 anti-phosphotyrosine antibody) that S597 was less potent than insulin in stimulating IR-A tyrosine phosphorylation. This discrepancy may arise from the use of different antibodies to detect total tyrosine phosphorylation (4G10 versus pY20), which are known in proteomic studies to pull down different subsets of phosphopeptides (39). Also, due to the nature of the KIRA assay, which involves immunocapturing the receptor with an anti-IR monoclonal antibody and detection with PY20, it is also possible that other tyrosine phosphorylated proteins associated with the IR-A are being detected. Whether this provides the basis for the difference in relative potency in each study remains to be explored. Notably, in our study of phosphorylation of individual tyrosine residues (p960, p3Y, p1318, and p1322, Figure 2; Table 2), S597 had a ~50% potency of insulin, which was unexpected from the binding affinity (Table 1) and total phosphorylation KIRA results (Figure 1) but was more in line with the total phosphorylation observation by Jensen et al. (16).

While the dose–response curves of IR-A activation mirrored the relative binding affinities, the time course revealed a significant difference between S597 and the other ligands. S597 induced a slow rate of activation, whereas the others ligands induced rapid phosphorylation and thereafter maintained a sustained response (Figure 3). Intriguingly, while Jensen et al. demonstrated that S597 supported sustained IR phosphorylation, they did not observe a slow rate of activation (16). Again, this could be a result of the different experimental approaches and at this stage it is unclear whether this is a cell line-specific effect. Jensen et al. (16) used L6 myoblasts overexpressing the IR-A [200,000 receptors/cell (40)] whereas R^−^ fibroblasts overexpressing the IR-A [50,000 receptors/cell (6)] were used in this study.

The mechanism underlying the gradual increase in receptor tyrosine phosphorylation promoted by S597 could be either a slow rate of the ligand association with the IR-A or the ligand-induced cross connection of two receptor halves (required for activation) occurring at a slow rate. De Meyts et al. have suggested that S597 dissociates from the receptor slowly [personal communication (29)], thus suggesting that the most likely explanation is that S597 may not promote the cross connection of the two receptor halves as efficiently as insulin (16, 41). The IR-A KIRA data described in the current study support this. Furthermore, while characterizing a library of peptide insulin mimetics from which S597 was developed, Pillutla et al. (30) suggested that there was a third ligand-binding site on the IR ectodomain, and suspected that it could be N-terminal to the ligand-binding site 2. In optimizing the receptor internalization assay, we determined that S597 competes with the anti-IR antibody 83-14 for binding to the IR-A (data not shown). As 83-14 binds N-terminal to the ligand binding site 2 (42), this observation supports the existence of a third site of interaction.

The observed differences in the pattern of total tyrosine and individual tyrosine residue phosphorylation upon activation by the different ligands suggests that binding of different ligands can lead to different phosphorylation patterns that influence subsequent biological actions. Hansen et al. (40) took a similar approach using antibodies to detect pY960, pY1146, and pY1322 in a study of insulin analogs. Interestingly, a preferential phosphorylation of Y960 over the other sites was promoted by the insulin analog X10 (B10Asp), which has a significantly higher mitogenic/metabolic ratio compared with insulin. pY960 acts as a docking site for SHC and IRS. SHC binding and activation leads to MAPK signaling and mitogenic actions, whereas activation of IRS proteins has a direct effect on the downstream Akt signaling pathway that plays a vital role in determining metabolic and mitogenic outcomes. Therefore, in the case of insulin X10, it is possible that the preferential Y960 phosphorylation plays a role in its increased mitogenic activity by altering downstream MAPK and/or Akt signaling. In our study, only Y1322 (and not Y960) appeared to be differentially phosphorylated in response to IGF-II and S597 (Table 2), although a more extensive dose–response experiment may reveal subtle differences for the other residues including Y960. Interestingly, Jensen et al. (16) reported that S597 was less potent than insulin in stimulating IRS-1 and IRS-2 phosphorylation and our data would suggest that this is due to the slow kinetics of Y960 phosphorylation (Figure 3B) that would then account for lower potency in Akt and MAPK activation (Figures 4C,D).

The kinetics of Akt (S473) and MAPK phosphorylation stimulated by insulin and IGF-II (Figures 4C,D) were similar to those reported by Sacco et al. (14), with Akt (S473) activation being sustained over the 30 min and MAPK phosphorylation being transient and maximal at 10 min. In the current study, IGF-II was significantly less potent than insulin in stimulating MAPK activation at the time of the maximal response (Figure 4D), but no difference between these two ligands was reported by Sacco et al. (14), possibly again reflecting a difference in cell lines used in the two studies. Interestingly, kinetic profiles of Y960, 3Y, Y1318, and Y1322 phosphorylation stimulated by all four ligands did not match the phosphorylation kinetics of Akt (sustained) and MAPK (transient), despite these phosphotyrosines being involved in recruitment of adapter molecules upstream of Akt and MAPK. The inability to correlate phosphorylation kinetics at Y960, 3Y, Y1318 and Y1322 with the kinetics of Akt and MAPK signaling most likely reflects the complexity of activation of downstream pathways, the interplay between kinases and phosphatases downstream of the receptor but also possibly that other tyrosine residues on the IR-A might be involved in promoting activation of these pathways.

In summary, insulin and qIGF-I have similar properties in IR-A binding and promotion of IR-A activation, internalization, and MAPK phosphorylation. Both promoted DNA synthesis to a similar extent. However, the relatively low affinity of IGF-II for IR-A and its lower potencies in inducing IR-A tyrosine phosphorylation and internalization compared to insulin resulted in reduced levels of MAPK phosphorylation and subsequent lower mitogenic potency (Figure 5B). In contrast, the dramatically different kinetics of receptor activation by S597 appeared to account for its inability to promote IR-A internalization, MAPK phosphorylation, and mitogenic activity [previously linked to an inability to repress Ccng2 (Cyclin G2) mRNA levels (43)]. None of the studied parameters could account for the reported lower metabolic activities of qIGF-I compared to insulin (33), suggesting the need for further investigation of the role of the other IR tyrosine residues and downstream signaling molecules. In addition, these results support the concept that not only does the strength of the interaction but also the specific molecular contacts between each ligand and the IR contribute toward the overall resultant biological outcomes.

## Conflict of Interest Statement

The authors declare that the research was conducted in the absence of any commercial or financial relationships that could be construed as a potential conflict of interest.
